# Supplementary material for: A Novel Quantitative Prediction Approach for Pungency Level of Chinese Liquor (Baijiu) Based on Infrared Thermal Imager
Source: Foods. 2021 May 17;10(5):1107. doi: 10.3390/foods10051107 (PMC8157151; doi:10.3390/foods10051107)

A novel quantitative prediction approach for pungency level of Chinese liquor (Baijiu) based on infrared thermal imager

Supplementary information

Table S1. The detail information of Baijiu samples used in the study

Sample	Alcohol (%, v/v)	pH	Total acid	Total ester (g/L)
S1	67.90	4.13	1.87	5.36
S2	67.50	4.01	1.85	5.28
S3	68.50	4.15	1.87	5.43
S4	68.80	4.14	1.88	5.36
S5	62.00	4.00	3.22	7.58
S6	60.90	4.02	2.66	5.32
S7	60.30	4.12	2.69	5.51
S8	63.70	4.11	2.67	5.89
S9	62.00	4.13	2.67	5.61
S10	61.20	3.84	2.67	5.49
S11	61.10	3.86	2.65	5.31
S12	61.00	3.85	2.59	5.25
S13	60.50	3.82	2.64	5.36
S14	64.00	4.43	1.88	5.29
S15	67.00	3.95	1.78	5.50
S16	67.10	4.01	1.78	5.57
S17	67.10	4.01	1.80	5.59
S18	64.60	3.92	2.08	5.66
S19	64.70	3.89	2.07	5.61
S20	65.10	3.91	2.07	5.68
S21	65.20	3.82	2.07	5.72
S22	62.10	3.87	0.66	5.87
S23	66.90	3.83	0.72	5.63
S24	67.90	3.93	0.69	6.72
S25	68.80	4.01	0.65	6.52
S26	68.80	4.01	0.78	5.69

Table S2. The temperatures of the tongue surface ROI 2 and the pungency intensity of Baijiu samples with different aging times.

Sample	T Min (℃)	T Max (℃)	T Aver (℃)	Pungency
S1	31.63±0.56	35.60±0.62	34.13±0.38	391.15±57.05
S2	31.58±0.29	35.80±0.59	33.80±0.54	317.44±36.03
S3	31.83±0.39	36.30±0.33	34.00±0.36	249.39±14.69
S4	31.95±0.53	36.07±0.62	34.07±0.28	296.28±20.00
S5	32.03±1.11	35.70±0.52	33.57±0.64	282.56±11.20
S6	31.63±1.06	35.93±0.92	34.13±0.21	349.08±29.35
S7	32.55±0.48	35.97±0.14	34.45±0.37	396.31±33.68
S8	32.58±0.59	36.17±0.47	34.35±0.48	395.43±29.51
S9	31.80±0.18	36.13±0.60	34.05±0.26	355.53±28.01
S10	31.73±0.82	35.50±0.75	34.27±0.25	356.39±25.86
S11	31.70±0.86	35.60±0.32	34.13±0.66	342.86±22.45
S12	32.03±1.40	36.13±0.72	34.47±1.07	380.44±61.58
S13	31.85±0.30	36.23±0.87	34.13±0.61	387.08±44.02
S14	32.50±1.04	36.77±0.57	34.55±0.57	403.67±29.74
S15	32.05±0.46	36.30±0.94	34.73±0.21	405.70±30.14
S16	31.90±1.09	35.73±0.75	34.27±0.47	400.80±56.37
S17	31.25±0.94	36.03±0.45	34.40±0.46	407.44±37.22
S18	32.73±1.03	37.20±1.10	35.20±0.46	471.31±31.83
S19	32.40±0.61	36.23±0.35	34.45±0.48	461.87±47.78
S20	31.85±0.45	36.33±0.35	34.20±0.16	391.12±26.04
S21	32.68±0.19	36.53±0.17	34.68±0.13	424.82±75.69
S22	31.38±0.68	36.10±1.01	33.93±0.29	374.71±37.86
S23	32.25±0.21	36.17±0.20	34.25±0.38	376.49±51.77
S24	31.75±1.02	35.75±0.69	34.27±0.55	432.50±55.64
S25	32.00±0.92	36.70±1.06	34.70±0.62	432.5±43.25
S26	32.40±0.75	36.10±0.54	34.53±0.41	408.92±38.43

-The temperatures of tongue surface ROI 2 were measured by the established IRT method with three selected subjects.
-The pungency intensity was evaluated by time-intensity method with 12 trained panelists and characterized with the area under the curve (AUC).

Table S3. The goodness of fit statistics of regression model

Statistic	Training set
Observations	22.000
Sum of weights	22.000
DF	18.000
R^2	0.648
Adjusted R^2	0.590
RMSE	31.506
F	11.065
Pr > F	0.000

Figure S1. Time-intensity curves of different Baijiu samples.

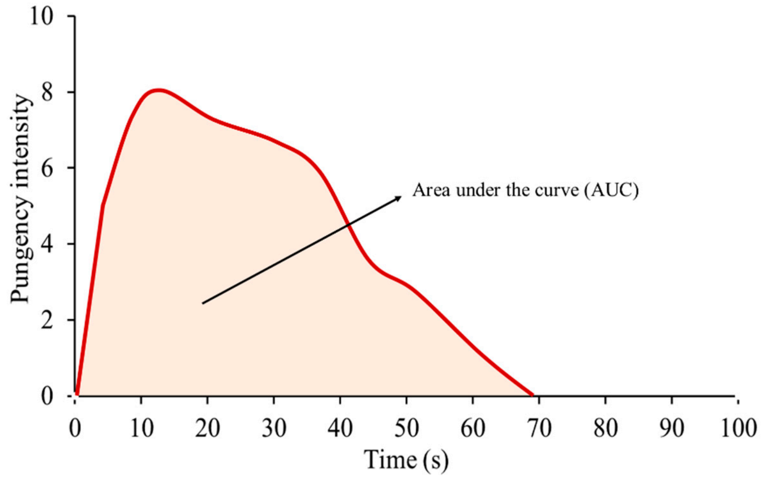

Supplement: Supplementary file 1 [file foods-10-01107-s001.zip › foods-1179923-supplementary.pdf]
